# BRefine: Achieving High-Quality Instance Segmentation

**DOI:** 10.3390/s22176499

**Published:** 2022-08-29

**Authors:** Jimin Yu, Xiankun Yang, Shangbo Zhou, Shougang Wang, Shangguo Hu

**Affiliations:** 1 College of Automation, Chongqing University of Posts and Telecommunications, Chongqing 400065, China; 2 College of Computer Science, Chongqing University, Chongqing 400044, China

**Keywords:** instance segmentation, sawtooth effect, segmentation inconsistency, rank and sort loss, boundary region loss

## Abstract

Instance segmentation has been developing rapidly in recent years. Mask R-CNN, a two-stage instance segmentation approach, has demonstrated exceptional performance. However, the masks are still very coarse. The downsampling operation of the backbone network and the ROIAlign layer loses much detailed information, especially from large targets. The sawtooth effect of the edge mask is caused by the lower resolution. A lesser percentage of boundary pixels leads to not-fine segmentation. In this paper, we propose a new method called Boundary Refine (BRefine) that achieves high-quality segmentation. This approach uses FCN as the foundation segmentation architecture, and forms a multistage fusion mask head with multistage fusion detail features to improve mask resolution. However, the FCN architecture causes inconsistencies in multiscale segmentation. BRank and sort loss (BR and S loss) is proposed to solve the problems of segmentation inconsistency and the difficulty of boundary segmentation. It is combined with rank and sort loss, and boundary region loss. BRefine can handle hard-to-partition boundaries and output high-quality masks. On the COCO, LVIS, and Cityscapes datasets, BRefine outperformed Mask R-CNN by 3.0, 4.2, and 3.5 AP, respectively. Furthermore, on the COCO dataset, the large objects improved by 5.0 AP.

## 1. Introduction

Instance segmentation is a classical task in computer vision that combines object-detection and semantic-segmentation tasks. It is widely used in fields such as unmanned vehicles and medical image analysis. HTC [1] designed a multitasking, multistage hybrid cascade structure that combines cascading and multitasking at each stage to improve information flow. It also incorporated a semantic segmentation branch to further improve accuracy. Fine boundaries, according to Cheng and others [2], can offer precise localization and improve the visibility of the mask segmentation. Object masks and boundaries are learned using the exemplary boundary information, and a mask head with preserved boundaries is built. Kirillov and others [3] viewed the image-segmentation problem as a rendering problem, and optimized object edge segmentation with a novel upsampling approach with better performance on edge segmentation. PointRend iteratively performs point-based predictions at blurred areas for high-quality image segmentation. CondInst [4] uses an instance-based dynamic instance-aware network instead of ROI, which lacks cropping and alignment operations, and speeds up inference. SOLO [5] transformed the instance segmentation problem into a category-aware prediction problem and an instance-aware mask-generation problem by dividing the grid and improving inference speed. YOLACT [6] generates instance masks with the linear combination of prototype masks and mask coefficients, and this process does not rely on repooling, which improves mask quality and inference speed. BlendMask [7] achieves high-quality mask prediction by combining top–down and bottom–up approaches to exploit fine-grained information at lower layers. Polytransform [8] is a postprocessing method that first generates instance-level masks using the segmentation network, and then transforms the masks into polygons and inputs them into the deformation network, which transforms these polygons into object boundary shapes.

**Figure 1:**
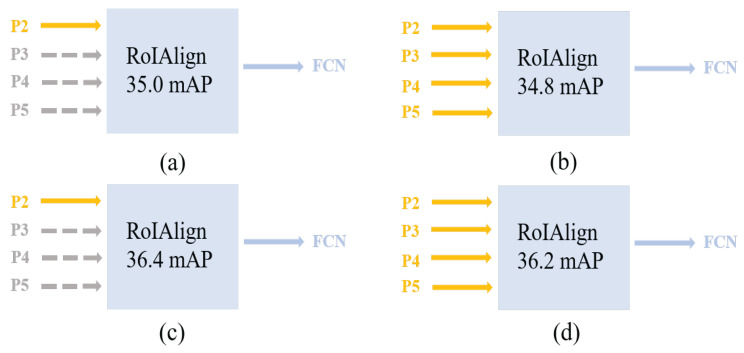
Comparison of mAP for different feature layers. P2, P3, P4 and P5 denote the output features of FPN [11]. Its output features are extracted by RoIAlign [9] and passed into the FCN [9]. (**a**,**c**) Extraction of the P2 feature layer of the FPN as the input features of the FCN. (**b**,**d**) Extraction of all its feature layers as input features to the mask head. (**a**,**b**) Resnet50 is used [12]. (**c**,**d**) Resnet101 is used [12]. On COCO2017 validation using a 1× training strategy, the above experiments were evaluated.

Mask R-CNN [9], a top–down detector that follows the idea of detection first and segmentation subsequently, is the most representative instance-segmentation approach. It uses a deep backbone network that drives the detector to obtain powerful localization and differentiation capabilities to recognize objects at different scales. However, deep networks result in coarse feature resolution. When these features are mapped back to the original input space, a large number of image details are lost. Feature alignment operation [9] further exacerbates this phenomenon. Unlike instance segmentation, semantic segmentation can gradually fuse shallow features through multiple upsampling operations to obtain high-resolution features with a large amount of detailed information, such as Unet [10]. Instance segmentation shares some traits with semantic segmentation.To prove this conjecture, the P2 feature map with the finest feature information in feature pyramid networks (FPNs) [11] is used as the input feature of the mask network. As shown in Figure 1, using the P2 layer as segmentation feature achieved the same performance as that using different layer features as segmentation features. This indicates that the P2 layer is fully equipped with different scales of mask information and has higher feature resolution.

**Figure 2:**
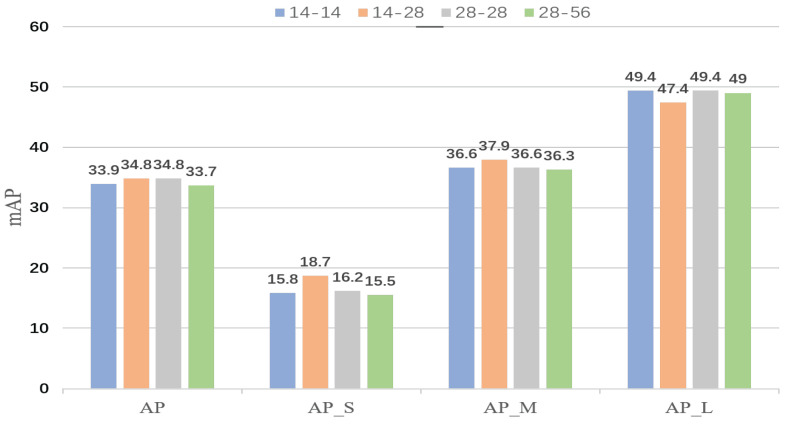
Comparison of the mask evaluation at different resolutions. The first 14 of 14-14 denotes that the input size of FCN is 14×14 and the second 14 denotes that its output size is 14×14. Different input and output were obtained in the same way. The above experiments used Resnet50 [12] as the backbone network, and were trained and validated on the COCO dataset using a 1× training plan.

The greater the resolution is, the more detailed the mask prediction in terms of feature space resolution. However, the experiment showed that the results were not so. With the change in resolution, inconsistency in segmentation appared at different scales. As shown in Figure 2, the performance effect of small- and medium-object segmentation decreases when the performance on large objects is improved. When the segmentation performance of large objects is poor, the segmentation performance of small and medium-sized objects is better. The loss function may be to blame for this phenomenon.To address this phenomenon, subsequent work will revolve around the loss function.

**Figure 3:**
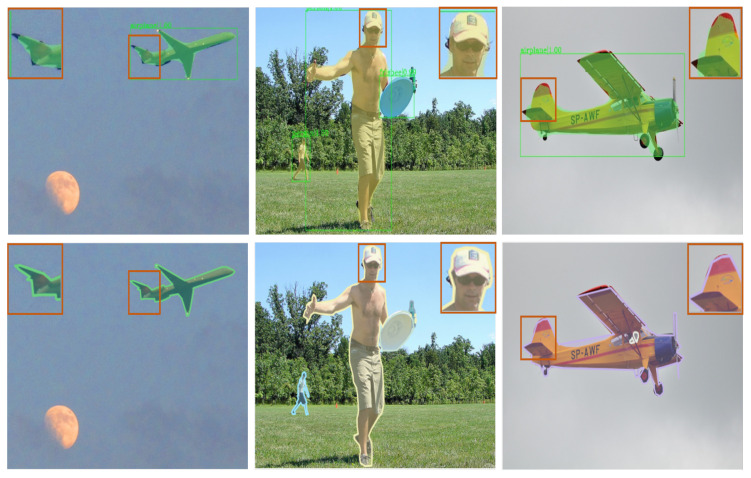
Display of predicted effects.The first row is the Mask R-CNN test sample. The second row is the labels.

The significance of the object’s boundary and shape information was ignored by previous instance segmentation methods [1,4,5,6,9,13,14], which treated all pixels equally. More consideration is given to object boundaries for a segmentation task. It is challenging to categorize the pixels of the boundary since the proportion of boundary pixels is significantly smaller than the proportion of overall object pixels (around 1% and even smaller for large targets). As shown in Figure 3, the boundaries are rough, and the overlap between objects is not reasonable. The prediction of the boundary pixels almost completely determines the segmentation quality. Fine boundaries, according to Cheng and others [2], can offer precise localization and improve the visibility of the mask segmentation. Object masks and boundaries are learned using the exemplary boundary information, and a mask head with preserved boundaries is built. Kirillov and others [3] viewed the image-segmentation problem as a rendering problem, and optimized object edge segmentation with a novel upsampling approach with better performance on edge segmentation.

On the basis of the analysis above, our primary goal was to build a straightforward and effective mask head that produces high-quality masks while retaining the robust detection capabilities of Mask R-CNN [9].To implement it, the FCN [9] mask-prediction network was used as the base network. The fine-grained mask features were then supplemented with much detailed information in the P2 layer. The detailed information that the model loses can be supplemented by these fine-grained features. The multistage idea is widely used in object detection [13,15] and image segmentation [1,3]. We applied this idea to instance segmentation to compensate for the loss of detailed features caused by ROIAlign. In particular, upsampling is used to gradually increase the 14×14 feature map to 56×56 using FCN as the baseline. Then, after convolutional layers, fine-grained features are gradually fused to produce high-quality mask prediction by parallelizing a fine-grained feature complimentary auxiliary line that extracts various resolution features on the P2 layer using RoIAlign. To address the difficulty of boundary partitioning, the cross-entropy loss function is extended, and boundary region cross-entropy loss (BRCE) is proposed. This loss function enables the model to put the focus on top of the boundary that is difficult to partition. Replacing the mask head with the proposed mask head, the multiscale segmentation inconsistency shown in Figure 2 occurs. Different resolutions may impact the cross-entropy loss function, which results in unstable segmentation. Balanced cross-entropy, focal [16], Dice [17], and their combinations of loss functions are used, and the effect is mitigated to some extent, but does not completely solve the problem. Due to the poor effect of a single loss function, multiobjective loss function was established.Rank and sort [18] loss (R and S loss) was introduced to solve the segmentation inconsistency. Boundary region cross-entropy loss was proposed to segment a finer boundary. By combining the two loss functions above, the BRank and Sort loss function is proposed. BRefine obtains significant results in segmentation tasks, especially in the target’s curved parts, and could obtain clear boundary masks.

We evaluated BRefine on different datasets and achieved significant segmentation results. Compared with Mask R-CNN, BRefine could output better segmentation quality, especially in difficult boundary regions. For large targets, the performance was improved by 5.0 AP.

## 2. Related Work

Instance segmentation. In recent years, the mainstream instance segmentation methods adopted a top–down segmentation method, that is, a powerful detector is used to generate a target frame, and then each pixel in the object frame is classified into the foreground and background. Deeper backbone networks are frequently used to enhance the performance of object detectors. This type of network, however, uses more downsampling operations, resulting in the loss of a large amount of image detail information. RoIAlign [9] performs scale normalization and feature extraction from the feature pyramid [11], which exacerbates the loss of image details and hinders producing high-quality instance masks. To obtain high-quality instance masks, instance segmentation is performed by supplementing detailed features.

Semantic segmentation. To supplement detailed information, the encoder–decoder structure of semantic segmentation increases the spatial resolution of the features. The renowned UNet [10] network joins the feature map of the encoder to the feature map of the decoder at each stage. The feature pyramid network (FPN) and ResNet network structure of the Mask R-CNN network resembles that of UNet [12]. The UNet network is different in that it only employs shallow features as segmentation features. The P2 layer provides rich mask information, as shown in Figure 1. Therefore, we used the P2 layer as a mask detail supplement feature. The detailed features of different resolutions are fused by a multistage approach. Loss function. Cross-entropy loss in segmentation tasks is susceptible to foreground and background pixels, favoring the side with more pixel points. Therefore, it requires a high balance of positive and negative pixels. Focal loss [16], proposed by He and others, addresses hard and easy samples, and positive and negative samples. On the basis of the cross-entropy loss function, we added the coefficients of positive and negative sample coefficients, and hard and easy sample coefficients. During the training process, the model focuses on samples in the priority order of positive hard, negative hard, positive easy, and negative easy. Dice [17] loss is a region-dependent loss function that, in semantic segmentation, primarily addresses the issue of extreme imbalance between positive and negative samples. In extreme circumstances, it may result in training instability. In addition to the above traditional loss functions, ranking-based loss functions directly optimize the performance metric, rendering the training and evaluation consistent, representing loss functions such as AP loss [19], and aLRP loss [20]. Such loss functions address classification and regression inconsistencies by concentrating more on positive than on negative samples. On the basis of these ranking-based loss functions, rank and sort loss [18] (R and S loss) is proposed. It further ranks the positive samples according to IoU and can address data imbalance. In addition, this function uses a heuristic algorithm to unify the multitask loss function.

## 3. Method

**Figure 4:**
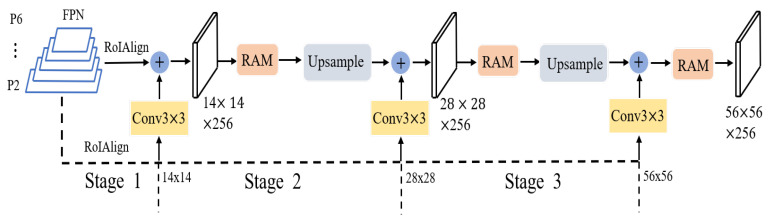
Framework for multiple-stage fusion. On the basis of FPN, different feature layers are extracted as the main line to provide deep semantic information. Parallel auxiliary lines with a fine-grained features complement the shallow detail information. The extracted detail features of different sizes are integrated with detail information by 3×3 convolution and then incorporated into the main line with deep semantic information. Each stage has a residual-aware module that obtains contextual information at different scales. These features are upsampled (bilinear interpolation) to gradually fuse higher-resolution detail information. To solve the segmentation inconsistency problem and the boundary pixel-scale imbalance problem, the BRank and Sort loss function is proposed. Higher mask quality is obtained.

An overview of BRefine is shown in Figure 4. BRefine performs high-quality instance segmentation on the basis of an FPN [11] with two feature-processing lines in parallel. One is the main line that obtains semantic information from different feature layers of the feature pyramid. The other is the auxiliary line that takes the highest resolution features in the feature pyramid as the most input and complements the detailed features. These fine features go through convolutional layers to obtain contextual information at that resolution. The mask head is executed in multiple stages. At each stage, it merges the semantic features with the fine features. Then, the contextual information at different scales is obtained through the residual perception module. BRank and Sort is proposed to render the model focused and bounded.

### 3.1. Multistage Fusion Mask Head

Mask R-CNN’s FCN mask head [9] was adopted as a baseline that provides multiscale semantic information (ROIAlign extracts ROIs in different feature layers with an initial resolution of 14×14). Fine-grained features (obtained from the P2 layer of features in FPN [11] with an initial resolution of 14×14) are supplemented in parallel with an auxiliary line. To obtain the fine-grained features’ contextual information, a convolutional layer is used on the extracted fine-grained features. The initial mask fusion features are then created by fusing the baseline semantic features with the auxiliary line fine-grained features. No additional processes are needed in this procedure, in keeping with the principles of simplicity and efficiency.

**Figure 5:**
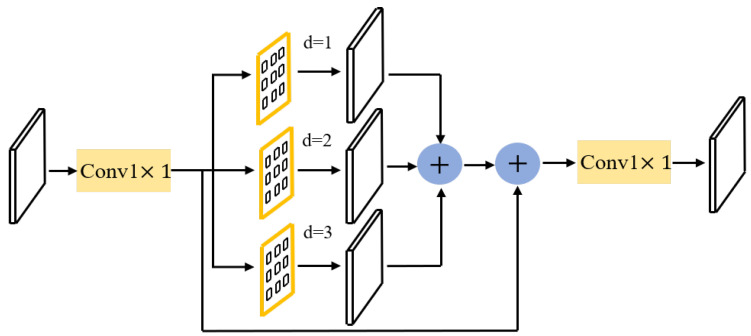
Residual-aware module: to extract varied-scale contextual information, the input features are first compressed by half through a 1×1 convolutional channel before being fed into three dilated convolutions (convolutional kernel is 3×3, and the dilated rates are 1, 2, and 3). A residual branch is paralleled, keeping the original resolution’s detailed information. These features are fused and then restored to the original channel after 1×1 convolution.

Following the aforementioned methods, the initial fusion features containing semantic and fine-grained features are obtained. To obtain more contextual information, a simple residual-aware module was designed. The fused features are first processed via a 1×1 convolutional compression channel to lessen the number of parameters, as shown in Figure 5. It is then fed into three parallel dilated convolutions to obtain different-scale contextual information. A residual branch is parallelized to keep the original local details. The obtained feature information is summed to obtain perceptual features with different scale information. The original feature channels are restored after 1×1 convolution. The features are upsampled to obtain high fine-grained features (bilinear interpolation is used here. The experiments showed that transposed convolution does not work as well as bilinear interpolation does). The previous step is repeated to lastly obtain high-resolution features with a resolution of 56×56 as our final prediction features. Higher-resolution features introduce more computational effort, which is not desirable. Therefore, we designed the resolution as follows.

### 3.2. Boundary Rank and Sort Loss

In combination with the cross-entropy loss function, a loss function based on the boundary region is proposed. This loss can automatically adjust the boundary width according to the image size. It enables the model to focus more on boundary regions that are difficult to partition due to more severe punishment. Multiscale segmentation inconsistency is a novel and thorny problem. R and S loss [18] solves the classification and regression inconsistency problem in detection tasks. The use of IoU as a classification label can solve the imbalance between positive and negative samples. It achieved excellent results in solving multiscale segmentation inconsistency. BR and S combines these two loss functions, and can achieve excellent performance in robustness and boundary segmentation.

**Figure 6:**
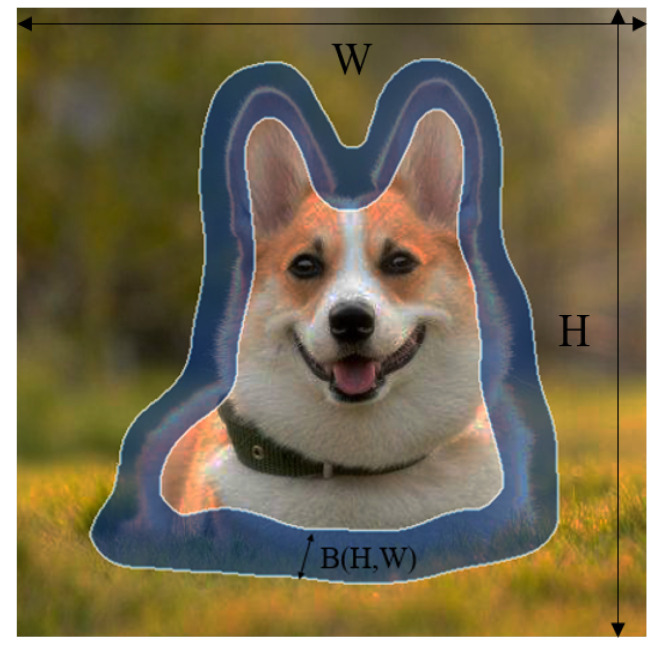
Boundary region: B(H, W) is the adaptive boundary are generated according to image size. The label’s width and height are W and H, respectively.

Boundary region loss: The erosion of labels using morphological principles. Rhis is a binary mask that is eroded (iterated according to image size) to obtain the erosion mask. The original mask minus the corrupted mask is regarded as the boundary mask (Figure 6). The formula is as follows:

(1) $$B_{k}\left(H,W\right)=G_{O}\left(H,W\right)-G_{E}\left(H,W\right)$$

where $G_{O}\left(H,W\right)$ denotes the original true mask, and $G_{E}\left(H,W\right)$ denotes the eroded mask after erosion of the original mask.

The obtained boundary region is combined with the cross-entropy loss function to propose the boundary region’s loss function. Its formula is as follows:

(2) $$L_{b}=\frac{1}{Z}\sum_{i\in K}\left(1+{\lambda B}_{i}\left(H,W\right)\right)CE\left(Y_{i},{\tilde{Y}}_{i}\right)$$

where Z denotes the number of samples, and K denotes the set of samples. The weight factor is 1.0 by default.$CE\left(Y_{k},{\tilde{Y}}_{k}\right)$ denotes the cross-entropy loss.

Boundary rank and sort loss: R and S loss [18] uses IOU as the optimization objective. The formula is as follows:

(3) $$L_{RS}=\frac{1}{Z}\sum_{i\epsilon (P\cup N)}\left(L\left(i\right)-L^{GT}\left(i\right)\right)$$

where the first item of $L\left(i\right)=\frac{{rank}^{-}\left(i\right)}{rank(i)}+\frac{\sum_{i\epsilon P}H\left(x_{ij}\right)\left(1-y_{j}\right)}{{rank}^{+}\left(i\right)}$ is the rank error, and the second item is the sort error. P is the positive sample set. N is the negative sample set. For error labels, rank error first expects all positive samples to be ranked before negative samples when the label value is 0. The sort error expecting only predicted scores with label scores larger than those of sample can be larger than itself, thus generating error. The label function equation is as follows:

(4) $$L^{GT}\left(i\right)=0+\frac{\sum_{j\epsilon P}H\left(x_{ij}\right)\left[y_{j}\geq y_{i}\right]\left(1-y_{j})\right.}{\sum_{j\epsilon P}H\left(x_{ij}\right)\left[y_{j}\geq y_{i}\right]}$$

Multitask loss function boundary R and S loss (BR and S) is proposed, combining the two loss functions above using a tuning strategy, which was formulated as follows:

(5) $$L_{BRandS}=\sum_{k=1}^{3}\left(L_{RS}^{k}+\lambda^{k}L^{k}\right)$$

where $L_{RS}^{k}$ is the R and S loss function for different tasks. $\lambda^{k}=L_{RS}^{k}/L^{k}$. $L^{k}$ is the average of the weighted sample loss, which is a weighting strategy based on the classification score. Its formula is as follows:

(6) $$L^{k}=\sum_{i\in P}\frac{w^{i}}{\sum_{j\in P}w^{j}}L^{k}$$

where P is the positive sample set. $w^{i}$ and $w^{j}$ are the sample classification scores for different tasks.

k=1 denotes RPN loss, where $w^{i}$ and $w^{j}$ are the RPN classification score. $L^{1}=wL_{GIoU}$. The default value of *w* is 0.2. $L_{GIoU}$ is GIoU loss [21].

k=2 denotes the loss of object detection, where $w^{i}$ and $w^{j}$ are the target detection classification score. $L^{2}=L_{GIoU}$. The inputs of $L_{RS}^{1}$ and $L_{RS}^{2}$ correspond to the IoU in RPN and the IoU in target detection, respectively.

k=3 denotes mask loss, where $L_{RS}^{3}=0$. $\lambda^{3}=L_{RS}^{2}/L^{3}$. $w^{i}$ and $w^{j}$ are the target detection classification score. $L^{3}=L_{b}$.

### 3.3. Experimental Details

We used Mask R-CNN as the baseline and replaced the default FCN mask head with the proposed multistage fusion mask head. The original multitask loss was replaced with the proposed BR and S loss to obtain the desired segmentation effect.

All experiments were implemented in MMDetection [22]. Due to the configuration of 3 RTX 3090 graphics cards, the learning rate for all model training was set to 0.0075. Except for the proposed novel approach, the hyperparameters were consistent with Mask R-CNN. Additionally, the ResNet50 [12] backbone network and the 1× learning strategy were used to train each model in the ablation experiment.

## 4. Experiment

To prove the effectiveness of the model, extensive experiments were performed on three datasets, namely, COCO [23], LVIS [24], and Cityscapes [25]. The standard mask evaluation provided by MMDetection [22] was ysed as the evaluation metric in the test experiments to ensure the uniformity of the evaluation criteria.

### 4.1. Main Results

The model performance was first tested at COCO 2017 using different backbones and different learning plans (Table 1). The performance of BRefine was much better than that of the baseline [9] while ensuring that other extraneous parameters were consistent. Adopting the ResNet50 [12] backbone, BRefine improved by 3.0 AP over the Mask R-CNN baseline, and by 5.0 AP for large-object evaluation. It still performed well under different training schedules.

### 4.2. Comparison with Previous Methods

On the COCO 2017 dataset, BRefine was compared with previous methods. The COCO dataset is a large-object detection and segmentation dataset that contains 80 categories, and features many categories and complex scenes. We trained the compared methods on train2017 and validated them on val2017. In the comparison experiments, a unified backbone network and a training plan were used to train different methods for comparison. Table 2 shows the COCO val2017 single-model performance comparison results used to compare with the previous methods. BRefine outperformed the previous model in most of the evaluated metrics. Since the used baseline is a top–down structure, the performance of upstream tasks affects the performance of downstream tasks. BRefine achieved superior results in masking even though the bbox performance metrics were weaker than HTC. This indicates that BRefine achieved more powerful segmentation performance.

### 4.3. Ablation Experiments

Extensive ablation experiments were performed on COCO val2017 to analyze the effectiveness of each part of BRefine. In the ablation experiments, a unified ResNet50 [12] backbone network was used along with a 1× training program (12 epochs). Except for the mentioned hyperparameters in the model, the remaining hyperparameters were kept consistent when not specifically stated.

**The effectiveness of the multistage fusion mask head.** The FCN mask head of Mask R-CNN was replaced with our proposed multistage mask head, and no residual-aware module was added here. As shown in Table 3, the more stages of fusion there were, the better the effect was, but the parameters showed exponential growth. Therefore, the number of stages was set to 3. The multistage fusion mask head could obtain better results for large objectives, but brought inconsistency in multiscale mask segmentation.

**The effectiveness of the residual-aware module.** The residual perception module was adapted to different stages to obtain different-scale contextual information. As shown in Table 4, after adding this module to obtain enough different-scale contextual information, the evaluation metrics were all effectively improved.

**The effectiveness of R and S loss.** As shown in Table 5, the introduction of this loss function caused a slight decrease in large-target segmentation, but a significant improvement in small- and medium-target segmentation. In particular, the small-target AP improved by 4.3 points. To further demonstrate the effectiveness of the multistage head in combination with R and S, the R and S loss function was used on the baseline [9]. Table 5 data show that the loss function achieved good performance improvement, but the combination of the multistage fusion mask had even better results. As a comparison, we show in the table the results using different loss functions.

**The effectiveness of boundary region loss.** Boundary area loss allows for the model to focus on those boundary pixels that are more difficult to focus on, improving model performance. As the object scale grows and the boundary pixels become fewer, the segmentation effect on large objects becomes increasingly obvious (Table 6).

### 4.4. Experiment on LVIS

The LVIS [24] dataset is long-tailed with large-scale fine-grained lexical tagging, and the annotation quality is higher than that of the COCO dataset to reflect the mask quality more accurately. The dataset contains 1203 categories with about 2 million high-quality instance segmentation annotations for the training, validation, and testing of images. The results are shown in Table 7, where BRefine improved AP by 4.2 points compared with the Mask R-CNN baseline. Due to the finer annotation, it was better than the COCO dataset on top of the segmentation effect.

### 4.5. Experimenting on Cityscapes

We also evaluated different models on the Cityscapes [25] dataset, which collects a variety of stereo video sequences recorded in street scenes from 50 different cities. In addition to containing 20,000 weak annotations, it contains 5000 frames of high-quality pixel-level annotations and 8 semantic classes for instance segmentation training, validation, and testing. As shown in Table 8, BRefine achieved superior performance.

### 4.6. Qualitative Results

**Figure 7:**
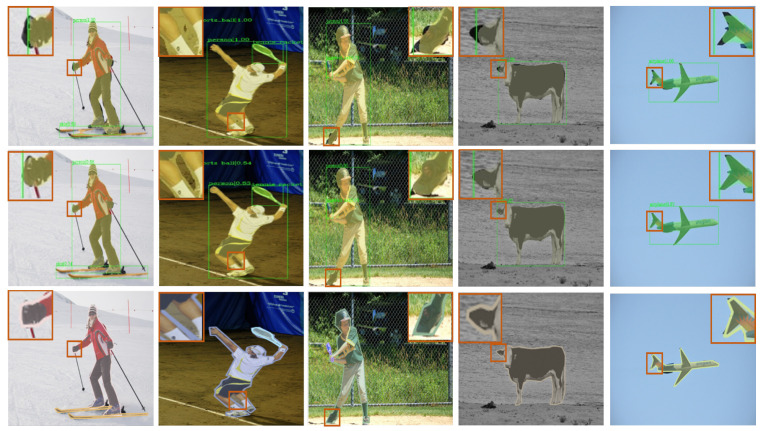
Visualization of the model on the COCO dataset. The first row indicates the Mask R-CNN test.

The model visualization on the COCO dataset is shown in Figure 7. The mask quality of BRefine was much larger than that of Mask R-CNN, especially for curve-change regions, such as the gloves that the person is wearing (first column) and the skeletonized region (second column). In some segmentation areas, the segmentation effect was better than labeling, such as the human shoulders (first column) and the tail of the machine (fourth column).

## 5. Discussion

In this work, we aimed to solve the mask coarseness problem in instance segmentation. The visualization (Figure 7) demonstrates that BRefine could output high-quality masks, especially in curved boundary areas to overcome polygon annotation defects. In comparison with previous methods (Table 2), BRefine achieved excellent performance.

However, BRefine still has limitations, mainly in the form of poor real-time performance (Table 2) and the lack of the interpretability of segmentation inconsistencies. Extracting the detailed information of objects at different scales on shallow features and higher output resolution features increases the computational cost, which results in poor real-time performance. The experiments (Table 5) show that the multiscale segmentation inconsistency is not caused by a single loss function, but by multitask losses. In a detection task, classification and regression are trained separately, and the loss is calculated and reverse-optimized. However, in prediction, it is filtered with classification scores. This may result in a bbox with high classification scores, but with bad regression being retained. Due to the top–down structure, feature maps are cropped using the bbox. The cropped feature maps are fed into the mask head. Thus, the segmentation task is directly influenced by the detection task.

Our future work will build on this foundation to design lightweight feature extractors that reduce computational cost and increase inference speed. We also aim to further explore the reasons for inconsistencies being generated in multiscale segmentation.

## 6. Conclusions

The research carried out in this paper introduced a high-quality image segmentation method based on deep learning. The method achieves high quality image segmentation through a simple and effective mask design with a better loss function. The overall results were better than those of other advanced instance segmentation algorithms, and they are summarized as follows.

1. The characteristics of the different feature layers of the FPN were analyzed in a segmentation task. Its lighter layer features had a different scale of mask information. On this basis, a multistage fusion mask head was proposed. The structure of this mask head was simple, but inconsistency in multiscale segmentation appeared. Having this problem in the FCN mask head architecture was experimentally found to be universal and a brand new problem.
2. Experimental data demonstrated that a single loss function cannot solve the inconsistency problem of segmentation. The multitask loss function of rank and sort can effectively solve this new problem. Despite solving this problem, there is still a lack of clear understanding and theoretical interpretability of this phenomenon. We will further investigate the root cause of this phenomenon.
3. The proposed boundary region loss function solved the problem of difficult boundary segmentation and achieved good segmentation results.

The BRefine model proposed in this paper has a simple structure and good segmentation effect, and can have broader application prospects in downstream tasks.

## Figures and Tables

**Table 1:** Comparison with Mask R-CNN on COCO val2017.

Method	Backbone	Schedules	AP	AP_S_	AP_M_	AP_L_	AP^bbox^
Mask R-CNN	R50-FPN	1×	34.8	18.7	37.9	47.4	38.3
BRefine	R50-FPN	1×	37.8	20.8	40.9	52.4	40.4
Mask R-CNN	R50-FPN	2×	35.5	18.9	38.9	48.5	38.8
BRefine	R50-FPN	2×	38.4	21.3	41.7	53.4	40.9
Mask R-CNN	R101-FPN	1×	36.2	19.1	40.0	49.5	40.1
BRefine	R101-FPN	1×	39.1	21.6	43.1	54.6	42.2
Mask R-CNN	R101-FPN	2×	36.7	19.6	40.6	51.5	40.2
BRefine	R101-FPN	2×	39.6	21.9	43.7	56.4	42.5

**Table 2:** Single-model comparison on COCO val2017. The above experiments were tested on 3 RTX 3090s using 1× training plans.

Method	Backbone	AP	AP_S_	AP_M_	AP_L_	AP^bbox^	fps
Mask R-CNN [9]	R50-FPN	34.8	18.7	37.9	47.4	38.3	24.6
Mask scoring [14]	R50-FPN	36.0	18.5	39.1	49.9	38.1	27.7
Bmask [2]	R50-FPN	36.1	19.1	40.8	50.9	-	-
HTC [1]	R50-FPN	37.3	19.4	40.2	51.3	41.9	8.2
CMask R-CNN [13]	R50-FPN	35.9	19.4	38.6	49.5	41.2	15.9
BRefine (ours)	R50-FPN	37.8	20.8	40.9	52.4	40.4	12.8
Point- prend [3]	R50-FPN	36.2	19.9	39.2	48.7	38.4	16.8
Blend- Mask [16]	R50-FPN	34.5	18.2	36.4	47.0	-	-
Yolact [6]	R50-FPN	28.9	11.3	32.5	43.4	31.2	42.3
Solo [5]	R50-FPN	33.1	12.2	36.1	50.8	-	-
CMask R-CNN	R101-FPN	37.3	19.7	40.6	51.5	42.9	14.1
Yolact	R101-FPN	30.4	12.0	33.9	46.2	33.1	36.4
HTC	R101-FPN	39.6	21.3	42.9	55.0	44.8	7.1
BRefine (ours)	R101-FPN	39.1	21.6	43.1	54.6	42.2	10.1
Mask R-CNN	R101-FPN	36.2	19.1	40.0	49.5	40.1	18.9
Mask Scoring	R101-FPN	37.7	19.8	41.4	52.3	40.4	19.2

**Table 3:** The effectiveness of the multistage head. The FCN mask head in Mask R-CNN was replaced with a multistage fusion mask head, and detailed experiments were performed for each stage.

Stages	Output Size	AP	AP_S_	AP_M_	AP_L_	AP^bbox^	Parameter
1	14 × 14	36.5	19.8	38.4	48.1	39.9	1.0 M
2	28 × 28	37.0	20.2	39.5	49.8	40.1	2.0 M
3	56 × 56	37.8	20.8	40.9	52.4	40.4	4.1 M

**Table 4:** Effectiveness of the residual-aware module. RAM denotes the residual-aware module. We conducted careful experiments on each part of the RAM. The dilated convolutions were increased in the order of dilated rates of 1, 2, and 3.

RAM	AP	AP_S_	AP_M_	AP_L_	AP^bbox^
1 single 3 × 3 Conv	36.2	19.4	38.4	49.1	39.8
2 parallel 3 × 3 Convs	36.7	19.8	39.2	50.2	40.0
3 parallel 3 × 3 Convs	37.1	20.2	39.9	51.1	40.2
3 parallel 3 × 3 Convs + Residual	37.8	20.8	40.9	52.4	40.4

**Table 5:** Effectiveness of rank and sort loss function. Multistage denotes the use of a designed mask head; multistage RS and RS Mask R-CNN denotes the combination of rank and sort loss function with the multistage mask head and with the baseline, respectively. In addition, the results of Focal [16], Dice [17] and their combinations applied to the mask head are shown.

Method	AP	AP_S_	AP_M_	AP_L_	AP^bbox^
Multistage	35.4	16.2	38.0	52.0	38.6
Dice	35.7	16.5	38.7	51.7	38.7
Focal	35.8	16.7	38.9	51.9	38.7
CE + Dice	36.0	16.9	38.9	51.8	38.9
RS + multistage	37.3	20.5	40.2	51.4	39.9

**Table 6:** The effectiveness of boundary region loss.

Method	AP	AP_S_	AP_M_	AP_L_	AP^bbox^
Multistage RS	37.3	20.5	40.2	51.4	39.9
BRefine	37.8	20.8	40.9	52.4	40.4

**Table 7:** Results on the LVISv1.0 validation set. All models were trained with a 1× schedule, and the hyperparameters were kept the same as those of MMDetection [22] except that the learning rate was set to 0.0075.

Method	Backbone	Schedules	AP	AP_r_	AP_c_	AP_f_	AP^bbox^
Mask R-CNN	R50-FPN	1×	21.7	9.6	20.9	27.9	22.5
BRefine	R50-FPN	1×	25.9	18.2	25.2	30.8	26.5

**Table 8:** Results on the Cityscapes validation set. All models were trained on an 8-epoch training schedule.

Method	Backbone	Schedules	AP	AP_S_	AP_M_	AP_L_	AP^bbox^
Mask R-CNN	R50-FPN	8-epoch	36.5	12.7	33.2	57.2	40.9
BRefine	R50-FPN	8-epoch	40.0	14.1	37.3	63.9	44.3

## Data Availability

Not applicable.
